# SNPer: An R Library for Quantitative Variant Analysis on Single Nucleotide Polymorphisms among Influenza Virus Populations

**DOI:** 10.1371/journal.pone.0122812

**Published:** 2015-04-13

**Authors:** Unitsa Sangket, Sukanya Vijasika, Hasnee Noh, Wasun Chantratita, Chonticha Klungthong, In Kyu Yoon, Stefan Fernandez, Wiriya Rutvisuttinunt

**Affiliations:** 1 Department of Molecular Biotechnology and Bioinformatics, Faculty of Science, Prince of Songkla University, Songkhla, Thailand; 2 Department of Pathology, Faculty of Medicine, Ramathibodhi Hospital, Mahidol University, Bangkok, Thailand; 3 Department of Virology, Armed Forces Research Institute of Medical Sciences, Bangkok, Thailand; Centers for Disease Control and Prevention, UNITED STATES

## Abstract

Influenza virus (IFV) can evolve rapidly leading to genetic drifts and shifts resulting in human and animal influenza epidemics and pandemics. The genetic shift that gave rise to the 2009 influenza A/H1N1 pandemic originated from a triple gene reassortment of avian, swine and human IFVs. More minor genetic alterations in genetic drift can lead to influenza drug resistance such as the H274Y mutation associated with oseltamivir resistance. Hence, a rapid tool to detect IFV mutations and the potential emergence of new virulent strains can better prepare us for seasonal influenza outbreaks as well as potential pandemics. Furthermore, identification of specific mutations by closely examining single nucleotide polymorphisms (SNPs) in IFV sequences is essential to classify potential genetic markers associated with potentially dangerous IFV phenotypes. In this study, we developed a novel R library called “SNPer” to analyze quantitative variants in SNPs among IFV subpopulations. The computational SNPer program was applied to three different subpopulations of published IFV genomic information. SNPer queried SNPs data and grouped the SNPs into (1) universal SNPs, (2) likely common SNPs, and (3) unique SNPs. SNPer outperformed manual visualization in terms of time and labor. SNPer took only three seconds with no errors in SNP comparison events compared with 40 hours with errors using manual visualization. The SNPer tool can accelerate the capacity to capture new and potentially dangerous IFV strains to mitigate future influenza outbreaks.

## Introduction

Influenza virus (IFV), a rapidly evolving virus in the orthomyxoviridae family, causes frequent epidemics and occasional pandemics. The diversity of the IFV genome generates mixtures of viral subpopulations, which subsequently can lead to the emergence of new virulent strains. Genetic divergence of IFV sequences can be driven by pressure from host immunity and host cell factors [1]. IFV evolves through several mechanisms including RNA recombination, point mutation or antigenic drift, and gene reassortment or genetic shift [2]. There are three types of IFVs (influenza A, B and C) with human disease most commonly caused by influenza A/H3N2, A/H1N1 and B. A full length RNA genome of influenza A is approximately 13.6 kb; influenza B is about 14.6 kb. The full genomic structure is composed of eight fragments with approximate lengths of 2341 nucleotides (nt) for RNA polymerase PB1 unit; 2300 nt for RNA polymerase PB2; 2233 nt for RNA polymerase PA; 1765 nt for hemagglutinin (HA); 1565 nt for nucleoprotein (NP); 1413 nt for neuraminidase (NA) with additional NB protein in influenza B; 1027 nt for matrix (M); 890 nt for nonstructural protein (NS) including NS1 and NS2 proteins.

Close monitoring of current circulating strains is crucial to evaluate IFV evolution as well as the possible detection of novel virulent and drug resistant viral strains. This information is essential for determining seasonal influenza vaccine design and composition. Therefore, a technique to identify the signature sequence or single nucleotide polymorphisms (SNPs) of viruses from the large amount of sequence information typically generated using next-generation sequencing (NGS) is essential to evaluate the sequence signatures in viral subpopulations associated with virulent or drug resistant phenotypes.

NGS [3], also known as high-throughput sequencing, is a powerful sequencing technique often used to obtain large amounts of genomic sequences to investigate specific questions about an organism’s genetic information. Particularly in viruses, this methodology has been adopted for diagnosis and advanced investigations to detect novel mutations and evolving quasispecies [4]. Analyzing SNPs by manual visualization is time consuming and resource intensive. A computational program to compare viral SNPs would provide an efficient tool compared to manual analysis.

R [5] is an open source statistics programming language and environment (http://www.r-project.org/). A wide variety of packages are provided by R, especially bioinformatics packages such as Bioconductor [6] (www.bioconductor.org), GenABEL [7] and ParallABEL [8] (http://www.genabel.org/packages). MySQL (http://www.mysql.com/) is a well known free database management software useful for storing and retrieving SNPs data using SQL command [9]. MySQL can be manipulated by R using RMySQL [10], a database interface and MySQL driver for R.

In this article, we present the development of the “SNPer” library, a new R library for identification of IFV mutations by differentiation of SNPs among viral subpopulations. “SNPer” is named to denote the action of searching for SNPs in a MySQL database.

## Methods

### Operating System and Computer Software for Input Data Preparation

A personal computer running CentOS (Community Enterprise Operating System) version 6.3 (http://www.centos.org/download/) was utilized for data analysis. The computer consisted of an Intel icore i5-2400 (3.10 GHz) processor and 4 GB RAM. This computer also provided R program version 3.0.2, RMySQL library version 0.9–3, and MySQL 5.1.67, which were utilized as components by the SNPer library.

SNPs data of three different influenza A/H3N2 viral subpopulations obtained by NGS and published in Sequence Read Archive (SRA) and GenBank [11–12] were used to create an input data file to measure the performance of SNPer. The IFV genomic information of NGS sequence read data as described in detail in Rutvisuttinunt et al. 2014 [12] including viral subpopulations from sample #VIROAF1 (GenBank accession number KJ577146-KJ577153), VIROAF2 (KJ577154-KJ577161) and VIROAF6 (KJ577186-KJ577193) were downloaded from the database (http://www.ncbi.nlm.nih.gov/sra and http://www.ncbi.nlm.nih.gov/nuccore/). Each subpopulation had eight fragments: PB2, PB1, PA, HA, NP, NA, M and NEP. The SNPs data of the three IFV subpopulations in csv files were created by Burrows-Wheeler Aligner (BWA) [13] and Genome Analysis Toolkit (GATK) [14–15] from the raw fastq files.

Table 1 illustrates an example of SNPs data of the HA gene fragment from VIROAF1 contained in “AF1_HA.csv” file. A *.csv file contained SNPs information of the sequence reads of interest after being aligned with the reference genome, and the fields of each line were separated by commas and enclosed within double quotation marks. The specific “AF1_HA.csv” file was created by MiSeq Reporter aligning sequence reads [13] with the NGS data of influenza A/H3N2 subpopulation 1 against the HA gene of the reference genome influenza A/H3N2 (GenBank CY121792). The Call column presents the alleles of the SNPs. For example, the first SNP at location 51 is A51G (VIROAF1 contains allele G while reference contains allele A at position 51 of HA gene fragment).

**Table 1:** Detected SNPs in HA gene fragment from sample #VIROAF1 (“AF1_HA.csv”).

**#**	**Sample ID**	**Sample Name**	**Chr**	**Position**	**Score**	**Variant Type**	**Call**	**Frequency**	**Depth**	**Filter**
**1**	**VIROAF1**	VIROAF1	H3N2_CY121792_HA.seq	**51**	13099	SNP	**A->[G/G]**	1	426	LowGQ
**2**	**VIROAF1**	VIROAF1	H3N2_CY121792_HA.seq	**72**	16707	SNP	**C->[T/T]**	1	567	LowGQ
**3**	**VIROAF1**	VIROAF1	H3N2_CY121792_HA.seq	**146**	23020	SNP	**A->[G/G]**	1	864	LowGQ
**4**	**VIROAF1**	VIROAF1	H3N2_CY121792_HA.seq	**182**	23194	SNP	**G->[A/A]**	1	872	LowGQ
**5**	**VIROAF1**	VIROAF1	H3N2_CY121792_HA.seq	**191**	23154	SNP	**C->[T/T]**	1	852	LowGQ
**6**	**VIROAF1**	VIROAF1	H3N2_CY121792_HA.seq	**285**	17047	SNP	**C->[T/T]**	1	620	LowGQ
**7**	**VIROAF1**	VIROAF1	H3N2_CY121792_HA.seq	**308**	16337	SNP	**T->[A/A]**	1	580	LowGQ
**8**	**VIROAF1**	VIROAF1	H3N2_CY121792_HA.seq	**405**	21306	SNP	**A->[G/G]**	1	791	LowGQ
**9**	**VIROAF1**	VIROAF1	H3N2_CY121792_HA.seq	**413**	20528	SNP	**G->[A/A]**	1	760	LowGQ
**10**	**VIROAF1**	VIROAF1	H3N2_CY121792_HA.seq	**456**	20446	SNP	**C->[T/T]**	1	768	LowGQ

Each line describes the SNPs at each position in the HA gene fragment of VIROAF1. For instance, position 51 of the HA gene in VIROAF1 is G while the reference allele is A.

### Executing SNPer

**Fig 1:**
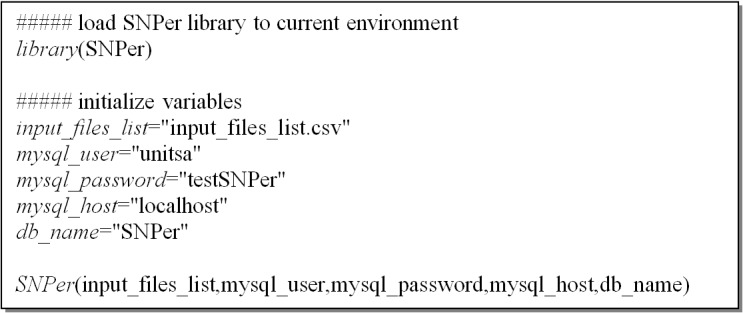
An example of SNPer usage. The user loads the SNPer library to current environment before using the SNPer function.

After installing the SNPer package, users can use SNPer to compare IFV SNPs by executing the SNPer function. An example of SNPer usage is shown in Fig 1. The user can change the variables including *input_files_list* (a file contained the list of SNPs files), *mysql_user* (a user name in MySQL), *mysql_password* (a password for the user name), *mysql_host* (the host name of MySQL), and *db_name* (the database name for storing SNPs data). The SNPer database must be created by the user before executing SNPer.

**Fig 2:**
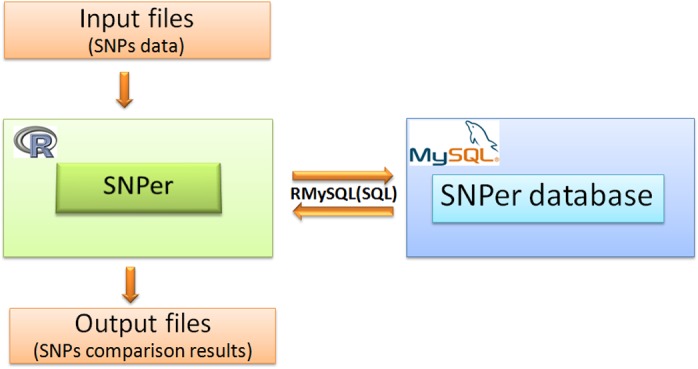
SNPs comparison workflow. SNPer, an R library, analyzes SNPs data using RMySQL and MySQL producing SNPs comparison data as its output.

The workflow for SNPs comparison is presented in Fig 2. The SNPs data in input files is processed by the SNPer library under the R program. SNPer uses RMySQL library and SQL commands to create three tables of the SNPer database including sp1, sp2 and sp3 [Table 2]. These tables store SNPs data of each viral subpopulation in the SNPer database. For instance, the sp1 table stores the SNPs data of the HA fragment of the first IFV subpopulation (VIROAF1) contained in “AF1_HA.csv” file, the sp2 table stores the SNPs data of the HA fragment of the second IFV subpopulation (VIROAF2) contained in “AF2_HA.csv” file, and the sp3 table stores the SNPs data of the HA fragment of the third IFV subpopulation (VIROAF6) contained in “AF6_HA.csv” file. SNPer executes data according to the list of subpopulations illustrated in Table 2. The SNPs data from “AF1_HA.csv”, “AF2_HA.csv” and “AF6_HA.csv” are pulled from the SNPer database for SNPs comparison by SNPer.

**Table 2:** List of SNP input files ("input_files_list.csv") to be compared by SNPer.

**sp1**	**sp2**	**sp3**
AF1_HA.csv	AF2_HA.csv	AF6_HA.csv
AF1_M.csv	AF2_M.csv	AF6_M.csv
AF1_NA.csv	AF2_NA.csv	AF6_NA.csv
AF1_NEP.csv	AF2_NEP.csv	AF6_NEP.csv
AF1_NP.csv	AF2_NP.csv	AF6_NP.csv
AF1_PA.csv	AF2_PA.csv	AF6_PA.csv
AF1_PB1.csv	AF2_PB1.csv	AF6_PB1.csv
AF1_PB2.csv	AF2_PB2.csv	AF6_PB2.csv

SNPs from eight fragments of sp1, sp 2 and sp3 were compared by SNPer. Each computational SNPs comparison among three viral subpopulations was conducted according to the name of the files listed in each row. For instance, for row #1, SNPer compared the SNPs data in file “AF1_HA.csv” of population 1 (sp1), “AF2_HA.csv” file from population 2 (sp2) and “AF6_HA.csv” file from population 3 (sp3).

In addition, the setting of RMySQL table structure for the input data is required as illustrated in Table 3 if different datasets are used for SNPs comparison. The input data (as seen in Table 1) contains information which is organized into eight fields, separated by columns in the csv file. The assigned primary key, “id” column, must be unique and not null. SNPer queries the SNPs comparison in the tables using RMySQL with SQL commands. Furthermore, SNPer is executed to compare the SNPs of three viral subpopulations ordered by the list of SNPs files as shown in Table 2. The SNPs comparison outputs from RMySQL are processed and written in the output files by SNPer.

**Table 3:** The required table structure of the input data for SNPer computational analysis based on MySQL.

**Field**	**Type**	**Null**	**Key**	**Default**	**Extra**
id	int(5)	NO	PRI	NULL	
sample_id	char(20)	YES		NULL	
sample_name	char(20)	YES		NULL	
chr	char(100)	YES		NULL	
position	int(20)	YES		NULL	
score	int(20)	YES		NULL	
variant_type	char(20)	YES		NULL	
call_	char(8)	YES		NULL	
frequency	int(5)	YES		NULL	
depth	int(10)	YES		NULL	
filter	char(20)	YES		NULL	

The table structure of the input data in the SNPer database can be retrieved by sql command (DESCRIBE <table_name>).

### Expected Output Data for SNPs comparison

**Fig 3:**
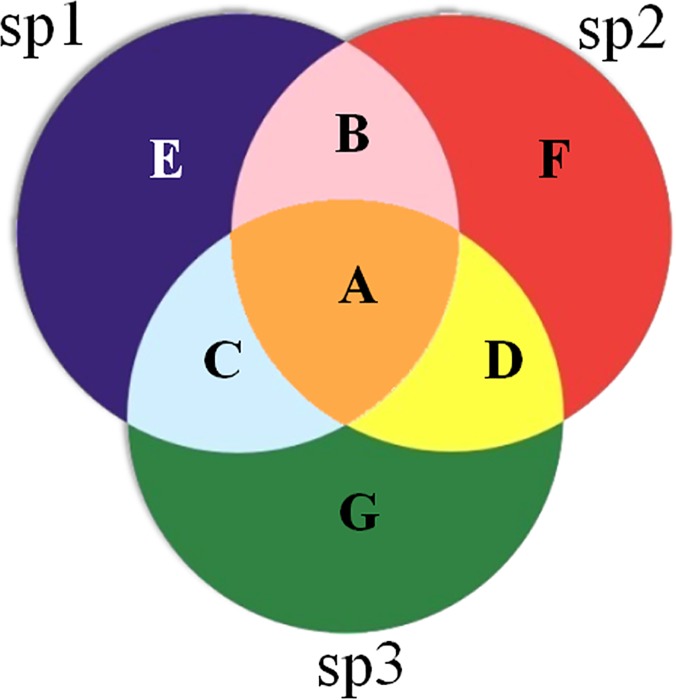
Comparison of SNPs from three different IFV subpopulations. Area A contains universal SNPs. Areas B, C and D consist of likely common SNPs. Areas E, F and G contain unique SNPs.

SNPer analyzes the output variants data from NGS done by the MiSeq Illumina platform and groups the SNPs of three different IFV subpopulations into (1) universal SNPs (shared in all viral subpopulations), (2) likely common SNPs (shared in almost all viral subpopulations), and (3) unique SNPs (not shared by other viral subpopulations). Fig 3 shows an example of SNPs comparison of three different viral subpopulations of influenza A/H3N2: viral subpopulation 1 (sp1), viral subpopulation 2 (sp2) and viral subpopulation 3 (sp3). Universal SNPs are located in area A. Likely common SNPs are presented in area B (in sp1 and sp2 but not in sp3), area C (in sp1 and sp3 but not in sp2) and area D (in sp2 and sp3 but not in sp1). Unique SNPs are indicated in area E (only in sp1), area F (only in sp2) and area G (only in sp3).

## Results

**Fig 4:**
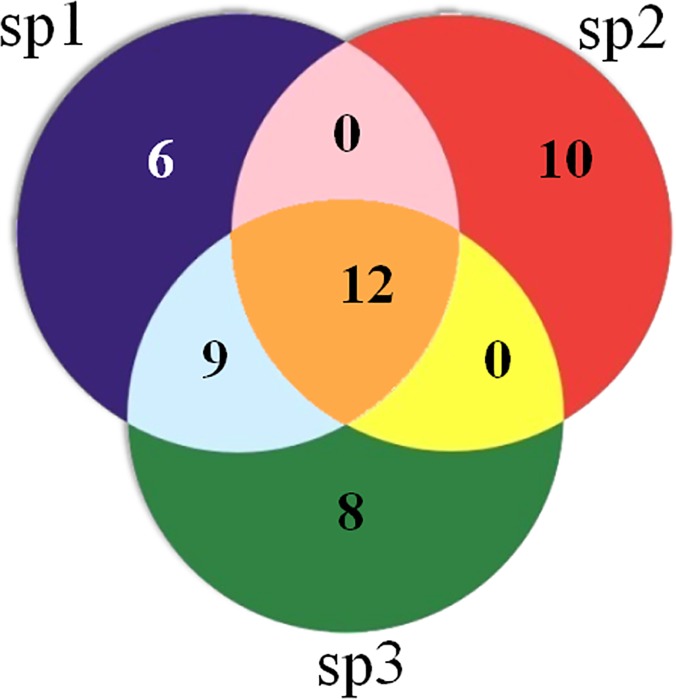
The SNPs composition output chart of three HA sequences from three IFV subpopulations. Each circle represents the number of SNPs of VIROAF1 (sp1), VIROAF2 (sp2) and VIROAF6 (sp3) for the HA fragment; universal SNPs; unique SNPs; and likely common SNPs.

SNPer took three seconds to compare the SNPs of the complete eight IFV genomic fragments against three viral subpopulations as shown in Table 4. Each row displays distinct groups of SNPs [unique (only_spX), likely common (only_spX_spY), and universal SNPs (all)]. The HA fragment has the highest number of universal SNPs (all = 12) and unique SNPs (only_X = 6 + 10 + 8 = 24) as visualized in Fig 4. The comparison results from SNPer were checked by adding groups of SNPs according to the formula; no errors were found using SNPer. The summation of the SNPs of all groups from SNPer was equal to the summation of the total SNPs numbers in the fragments of the three subpopulations.

**Table 4:** The outputs of SNPer for the eight fragments of the three different IFVs (“summary.csv”).

**samples_table**	**sp1**	**sp2**	**sp3**	**all**	**only_sp1**	**only_sp2**	**only_sp3**	**only_sp1_sp2**	**only_sp2_sp3**	**only_sp3_sp1**
AF1_HA_vs_AF2_HA_vs_AF6_HA	27	22	29	12	6	10	8	0	0	9
AF1_M_vs_AF2_M_vs_AF6_M	8	6	7	4	1	2	0	0	0	3
AF1_NA_vs_AF2_NA_vs_AF6_NA	13	12	16	5	3	6	5	0	1	5
AF1_NEP_vs_AF2_NEP_vs_AF6_NEP	5	4	5	2	0	2	0	0	0	3
AF1_NP_vs_AF2_NP_vs_AF6_NP	11	9	10	3	3	6	2	0	0	5
AF1_PA_vs_AF2_PA_vs_AF6_PA	15	8	18	3	3	5	6	0	0	9
AF1_PB1_vs_AF2_PB1_vs_AF6_PB1	18	21	21	9	3	11	5	0	1	6
AF1_PB2_vs_AF2_PB2_vs_AF6_PB2	31	16	26	11	7	5	2	0	0	13

Each row shows the number of SNPs of VIROAF1 (sp1), VIROAF2 (sp2) and VIROAF6 (sp3) for each fragment after comparison by SNPer. For example, in the first row, the HA fragment contains 27 SNPs in VIROAF1, 22 SNPs in VIROAF2 and 29 SNPs in VIROAF6. Twelve universal SNPs are in VIROAF1, VIROAF2, and VIROAF6. There are six SNPs in only VIROAF1, 10 SNPs in only VIROAF2 and eight SNPs in only VIROAF6. Only nine SNPs exist in both VIROAF6 and VIROAF1.(1)

sum=only_sp1+only_sp2+only_sp3+(only_sp1_sp2+only_sp2_sp3+only_sp3_sp1)*2+all*3 (1)

***only_spX*:** number of SNPs only detected in the X subpopulation.
***only_spX_spY*:** number of SNPs shared between the X and Y but not Z subpopulation.
***all***: number of SNPs shared by all subpopulations (X, Y and Z).

For example, the results from SNPer for the HA fragment of viral subpopulations VIROAF1, VIROAF2, and VIROAF6 were validated with the number of SNPs from Table 3. The summation of the number of SNPs in the HA fragment of the three subpopulations is 78 (27+22+29). The summation of SNPs from each group for the HA fragment of the three subpopulations is 78 [6+10+8+(0+0+9)*2+(12*3)]. Therefore, SNPer correctly produced the outputs of the HA fragment of the three subpopulations.

The complete list of SNPs for each group is provided in an output folder. An example of the list is shown in Table 5. The position and call (e.g., A405G) illustrates the universal SNPs from the three subpopulations.

**Table 5:** The allelic list of the universal SNPs found in HA fragment (“AF1_HA_vs_AF2_HA_vs_AF3_HA_sim_all.csv”).

id	sample_id	sample_name	id	sample_id	sample_name	id	sample_id	sample_name	position	call_
8	VIROAF1	VIROAF1	5	VIROAF2	VIROAF2	6	VIROAF6	VIROAF6	405	A->[G/G]
9	VIROAF1	VIROAF1	6	VIROAF2	VIROAF2	7	VIROAF6	VIROAF6	413	G->[A/A]
11	VIROAF1	VIROAF1	9	VIROAF2	VIROAF2	11	VIROAF6	VIROAF6	482	A->[G/G]
13	VIROAF1	VIROAF1	10	VIROAF2	VIROAF2	14	VIROAF6	VIROAF6	629	C->[T/T]
14	VIROAF1	VIROAF1	11	VIROAF2	VIROAF2	15	VIROAF6	VIROAF6	640	G->[T/T]
17	VIROAF1	VIROAF1	13	VIROAF2	VIROAF2	16	VIROAF6	VIROAF6	715	G->[A/A]
19	VIROAF1	VIROAF1	16	VIROAF2	VIROAF2	21	VIROAF6	VIROAF6	973	A->[G/G]
21	VIROAF1	VIROAF1	17	VIROAF2	VIROAF2	22	VIROAF6	VIROAF6	1195	A->[C/C]
23	VIROAF1	VIROAF1	18	VIROAF2	VIROAF2	25	VIROAF6	VIROAF6	1323	A->[G/G]
24	VIROAF1	VIROAF1	19	VIROAF2	VIROAF2	26	VIROAF6	VIROAF6	1341	T->[G/G]
26	VIROAF1	VIROAF1	21	VIROAF2	VIROAF2	28	VIROAF6	VIROAF6	1606	C->[T/T]
27	VIROAF1	VIROAF1	22	VIROAF2	VIROAF2	29	VIROAF6	VIROAF6	1671	A->[G/G]

Twelve universal SNPs detected among three subpopulations in HA fragment are A405G, G413A, A482G, C629T, G640T, G715A, A973G, A1195C, A1323G, C1606T, A1671G when compared to the influenza A/H3N2 reference (GenBank CY121792). Each row displays each SNP position.

## Discussion and Conclusion

The SNPer library utilizes RMySQL to compare IFV SNPs stored in MySQL. SNPer was efficiently executed on Linux and Microsoft Windows operating systems. In addition, manual visualization utilizing the same set of SNPs data by qualified performers under non-distracting conditions generally required more than 40 hours (data not shown).

Although similar software packages already exist, SNPer has certain advantages compared to currently available package tools. For instance, VCFtools [16] can compare two subpopulations (two files) whereas SNPer can compare three subpopulations. Although the VCFtools user can merge the SNPs data of two subpopulations to compare with a third subpopulation, it is a cumbersome and time-consuming process, especially when running many subpopulation fragments. A second software package, VariantToolChest [17], requires reference genomes in fasta format, and is time and memory consuming. In contrast, SNPer does not need any reference genome during comparison and takes less time and memory when compared to VariantToolsChest. Moreover, the VariantToolsChest user needs to create many different sets of commands to compare the fragments from three subpopulations. For example, to compare the SNPs of eight gene fragments from three subpopulations, VariantToolsChest requires 56 different commands, while SNPer requires one command. Therefore, SNPer is more efficient than VariantToolsChest, especially during analysis of multiple genomic fragments. In addition, users validate the outcome from VariantToolsChest and VCFtools whereas sql commands automatically validate outcomes from SNPer. In terms of running time and the resources needed to analyze our test dataset, SNPer requires the least, followed by VCFtools and VariantToolsChest.

In conclusion, SNPer is a rapid and efficient tool to detect SNPs to monitor IFV evolution. This efficiency only increases with higher numbers of SNPs. SNPer could be used to analyze quantitative variants of SNPs among not only IFV subpopulations [12] but also other pathogens such as human immunodeficiency virus (HIV). SNPer has the potential to improve our ability to understand evolving populations of viruses and other pathogens, particularly for identifying novel universal SNPs associated with specific traits (e.g., drug resistance, virulence, etc.) which can emerge under selective pressure. This tool could allow for more timely response to these newly emerging pathogens.

### Software Availability

The SNPer package and its manual are available at http://www.mbb.psu.ac.th/SNPer/index.html
